# Air Pollution and Preterm Birth: A Scoping Review Focused on Preterm Birth Phenotype and Specific Lengths of Gestation

**DOI:** 10.3390/children13010002

**Published:** 2025-12-19

**Authors:** Lindsey Abellard, Vy Le, Timothy D. Nelin, Sara B. DeMauro, Kristan Scott, Jane E. Clougherty, Heather H. Burris

**Affiliations:** 1 Department of Biostatistics, Epidemiology and Informatics, Perelman School of Medicine, University of Pennsylvania, Philadelphia, PA 19104, USA; lindsey.abellard@pennmedicine.upenn.edu; 2 Perelman School of Medicine, University of Pennsylvania, Philadelphia, PA 19104, USA; vy.le2@pennmedicine.upenn.edu; 3 Department of Pediatrics, Perelman School of Medicine, University of Pennsylvania, Philadelphia, PA 19104, USA; nelint@chop.edu (T.D.N.); demauro@chop.edu (S.B.D.); scottk8@chop.edu (K.S.); 4 Division of Neonatology, Department of Pediatrics, Children’s Hospital of Philadelphia, Philadelphia, PA 19104, USA; 5 Center of Excellence in Environmental Toxicology, Perelman School of Medicine, University of Pennsylvania, Philadelphia, PA 19104, USA; 6 Leonard Davis Institute of Health Economics, Philadelphia, PA 19104, USA; 7 Department of Environmental and Occupational Health, Dornsife School of Public Health, Drexel University, Philadelphia, PA 19104, USA; jec373@drexel.edu; 8 Department of Obstetrics and Gynecology, Perelman School of Medicine, University of Pennsylvania, Philadelphia, PA 19104, USA

**Keywords:** Preterm birth, spontaneous preterm birth, medically indicated preterm birth, air pollution, PM_2.5_, NO_2_

## Abstract

**Highlights:**

**What are the main findings?**

• While there have been over 100 studies of ambient PM_2.5_ and NO_2_ with preterm birth since 2011, only one in four studies reports on specific lengths of gestation.
• Even fewer (one in fifteen) report on specific preterm birth phenotypes (i.e., spontaneous or medically indicated).

**What are the implications of the main finding?**

• Preterm birth is heterogeneous with respect to lengths of gestation as well as the etiology, but is often analyzed as a single outcome in environmental health studies.
• Future studies of air pollution and other environmental exposures with preterm birth should disaggregate preterm birth phenotypes to shed light on potential mechanisms and to focus prevention strategies.

**Abstract:**

**Background/Objectives:** Air pollution is a recognized risk factor for preterm birth (PTB), a major cause of neonatal morbidity and mortality. The biological mechanisms underlying this association remain unclear, partly because PTB is a composite outcome that includes both spontaneous (sPTB, from preterm labor or rupture of membranes) and medically indicated (mPTB, for conditions such as preeclampsia or fetal growth restriction) subtypes. Additionally, PTB spans a range of gestational lengths from 20 to 36 completed weeks, which may reflect distinct etiologic pathways. **Methods:** This scoping review identified studies evaluating two pollutants strongly linked to PTB—particulate matter < 2.5 µm in diameter (PM_2.5_) and nitrogen dioxide (NO_2_)—in relation to PTB phenotypes and gestational length. A comprehensive PubMed search using targeted MeSH terms and keywords included studies published between 1 January 2011 and 28 February 2024. Eligible studies examined associations of PM_2.5_ or NO_2_ with PTB and were categorized by whether they specified PTB phenotype (sPTB or mPTB), gestational length, or neither. **Results:** Of 436 eligible studies, 5 evaluated specific PTB phenotypes, 28 considered gestational length, and 3 addressed both. Reported associations of PM_2.5_ or NO_2_ with PTB were frequently significant but varied in magnitude and direction. **Conclusions:** Few studies have examined pollutant exposure in relation to PTB phenotypes or gestational lengths, revealing an important knowledge gap. Standardized approaches to exposure assessment and PTB classification are needed to clarify causal pathways and inform targeted prevention strategies and policies to reduce pollution-related PTB.

## 1. Introduction

Air pollution represents an important macro-environmental exposure associated with several health outcomes, including adverse pregnancy outcomes. Air pollution is a complex mixture of gases, chemicals, and particulate matter emitted through natural processes and human activities such as fossil fuel combustion. Two measures of air pollution, particulate matter < 2.5 microns in diameter (PM_2.5_) and nitrogen dioxide (NO_2_), have been consistently linked to preterm birth (PTB), a major contributor to infant morbidity and mortality [1]. Based on these data, the American College of Obstetricians and Gynecologists and the American Academy of Pediatrics have released statements regarding the impact of environmental factors, especially ambient air pollution, on perinatal health outcomes [2,3].

Despite the widespread recognition of the harms of air pollution on perinatal outcomes, mechanisms linking air pollution exposure to PTB are unknown. Discovering mechanisms by which pollution contributes to PTB risk is especially challenging because PTB is a composite phenotype that can range from spontaneous (sPTB), from preterm labor or rupture of membranes, to medically indicated (mPTB), for conditions such as severe preeclampsia or impaired fetal growth [4]. Furthermore, PTB is heterogeneous with respect to lengths of gestation, ranging from 20 to 36 completed weeks, and is commonly classified as moderate to late preterm (32 to <37 weeks), very preterm (28 to <32 weeks), and extremely preterm (<28 weeks) [5]. Due to variation in the prevalence of morbidities leading into pregnancy, populations are more or less susceptible to conditions that may lead to mPTB due to maternal health (e.g., hypertensive disorder of pregnancy) or fetal health (e.g., fetal growth restriction). Due to differences in exposure and susceptibility, air pollution exposure–outcome associations may vary [6].

Taken together, these methodological challenges underscore that pregnancy is a uniquely time-limited period characterized by substantial cohort attrition and multifaceted risk, making rigorous and bias-aware approaches essential in perinatal research [7]. Given the variability in PTB phenotypes and lengths of gestation, this scoping review synthesizes existing evidence on associations of PM_2.5_ and NO_2_ with PTB phenotype and lengths of gestation.

## 2. Methods

### 2.1. Scope of Review

We evaluated studies examining associations of PM_2.5_ and NO_2_ exposure with PTB phenotypes and specific lengths of PTB gestation. We followed the Preferred Reporting Items for Systematic Reviews and Meta-Analyses (PRISMA) guidelines.

### 2.2. Research Question

What proportion of studies investigating associations of prenatal exposure to PM_2.5_ or NO_2_ with PTB consider phenotypes or specific lengths of gestation?

### 2.3. Eligibility Criteria and Study Selection

Studies published between 1 January 2011 and 28 February 2024 were deemed eligible for inclusion. Stieb et al. published a comprehensive systematic review and meta-analysis of ambient air pollution, birth weight, and PTB in 2012, including studies published through January 2011 [8]. We were specifically interested in the more recent literature and its focus, or lack thereof, on PTB phenotypes and specific lengths of gestation, and chose to include only studies after the Stieb review. Studies that included either PM_2.5_ or NO_2_ and the outcome of PTB were included for abstract and title review. A literature search was performed using PubMed to identify studies published in English. We used the following syntax and Medical Subject Headings (MeSH) terms for our search: (“PM_2.5_” [All Fields] OR “NO_2_” [All Fields] OR “Particulate matter” [MeSH Terms] OR “Air Pollution” [MeSH Terms:noexp]) AND (“Premature Birth” [MeSH Terms] OR “infant, premature” [MeSH Terms] OR “preterm delivery” [All Fields] or “preterm birth” [All Fields]). There were no sample size or geographic inclusion or exclusion criteria. Two independent research assistants (LA, VL) performed a title and abstract review followed by a preliminary full-text review, excluding review articles, articles that investigated non-human subjects, articles that did not include PM_2.5_ or NO_2_, and articles that did not include PTB as an outcome. Secondary, full-text review identified articles that specified PTB phenotype or specific lengths of gestation as outcome measures. Any discrepancies in study selection were resolved through consensus and reviewed by a neonatologist (TDN). Of note, we did not mandate the use of singular definitions for specific lengths of gestation, such as the World Health Organization categories of extremely, very, and moderate to late PTB. Instead, we included manuscripts with any specific distinctions made among various lengths of gestation.

### 2.4. Study Categorization

The primary outcome was whether a study evaluated either PTB phenotype or specific lengths of gestation. We categorized articles based on the exposures used and the outcomes measured. We then classified each study as positive, negative, null, or mixed with respect to results and reported whether they evaluated specific windows of exposure, such as trimester-specific associations.

## 3. Results

**Figure 1:**
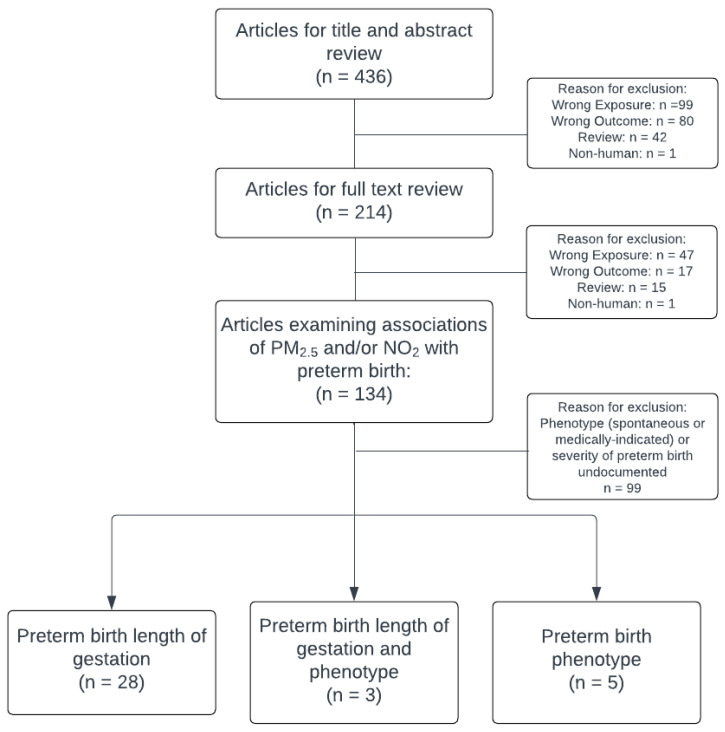
Flowchart for study inclusion.

We identified 436 eligible articles that met our search criteria within the study period (Figure 1). We excluded 222 articles in title and abstract review for being a review (*n* = 42), focusing on non-human subjects (*n* = 1), or including the wrong exposure (*n* = 99) or outcome (*n* = 80). We further excluded 80 articles in preliminary full text review for being a review (*n* = 15), non-human subjects research (*n* = 1), or including the wrong exposure (*n* = 47) or outcome (*n* = 17). Of the remaining 134 studies, 36 (26.9%) included PTB phenotype or specific lengths of gestation; five reported on phenotype alone, 28 reported on lengths of gestation alone, and three reported on both phenotype and lengths of gestation. Given the substantial difference in PM_2.5_ and NO_2_ exposures between studies conducted in China and those conducted elsewhere, results are presented separately by geographic location in each sub-section below.

### 3.1. Studies Reporting PTB Phenotype (sPTB or mPTB)

Table 1, Table 2 and Table 3 display studies that analyzed PTB phenotype (*n* = 5 without specific lengths of gestation) [9,10,11,12,13]. Studies of PM_2.5_ in Australia, Israel, New York City and Philadelphia were largely null with respect to sPTB or mPTB, with the exception of one study in Victoria, Australia that reported 4% higher risk of sPTB per IQR increment of PM_2.5_ exposure. The three studies of NO_2_ with PTB phenotype were outside of China and reported no increased risk (Table 1).

A cohort of 1515 twins in Shanghai, China reported that PM_2.5_ exposure was associated with 48% higher odds of sPTB per interquartile range (IQR) increase in PM_2.5_ during the second semester (OR 1.48; 95% CI: 1.06, 2.05) while associations in the first and third trimesters and entire pregnancy were null (Table 3) [12]. In another study of 179,385 singleton births in Shanghai, China, PM_2.5_ exposure in the third trimester had the strongest and most consistent association with sPTB (aOR 1.042; 95% CI: 1.018, 1.065) [13].

### 3.2. Studies Reporting Positive Findings with PTB Specific Lengths of Gestation

Table 4, Table 5, Table 6 and Table 7 display studies that measured specific lengths of gestation (*n* = 28, without PTB phenotype) [17,18,19,20,21,22,23,24,25,26,27,28,29,30,31,32,33,34,35,36,37,38,39,40,41,42,43,44]. There were 12 studies that consistently found positive associations of PM_2.5_ or NO_2_ exposure with specific lengths of gestation. Often, effect estimates were strongest for very PTB.

### 3.3. Studies Reporting Positive Findings with Specific Lengths of Gestation—PM_2.5_

Sheridan et al. analyzed over two million births in California and positive associations with PTB (HR 1.12; 95% CI: 1.09, 1.14), moderate PTB (HR 1.11; 95% CI: 1.09, 1.14), and very PTB (HR 1.19; 95% CI: 1.14, 1.25) per 10 μg/m^3^ increment of PM_2.5_ exposure over the entire pregnancy (Table 4) [19]. A study in Florida also reported a positive association; each IQR increment of PM_2.5_ exposure over the entire pregnancy was associated with very PTB (aOR 1.082; 95% CI: 1.048, 1.117) [22]. A nested case–control population in California observed slightly stronger associations of PM_2.5_ with moderate PTB compared to very PTB [26]. In an ecological study of 5 million births in France, associations of 7-year averaged, spatially resolved PM_2.5_ exposure were analyzed with PTB length of gestation revealing moderate positive associations with very early and extreme PTB [30]. In a time-stratified case-crossover study investigating short-term increases in PM_2.5_ during the cold season (November–April) in 196,970 singleton pregnancies in San Joaquin Valley, CA, per IQR increment, PM_2.5_ exposure was associated with a 5–6% increased odds of very PTB beginning 3 days prior to birth (lag 3), but no significant associations were detected with moderate PTB [17].

A retrospective cohort in Henan Province, China observed a 9.8% increase in early PTB < 34 weeks’ gestation and an 18.3% increase in late PTB between 34 and 36 weeks’ gestation per standard deviation increment of PM_2.5_ exposure (Table 6) [47]. A study of 2101 births in Wuhan, China also found that the strongest associations were with very PTB [47]. Another Chinese study of more than 2 million births reported that third trimester exposures had the strongest association with very and moderate PTB [21,22]. In contrast, another nationwide study of singleton births in China found that PM_2.5_ exposure was most associated with late PTB, followed by moderate PTB, and very PTB [36].

### 3.4. Studies Reporting Positive Findings with Specific PTB Lengths of Gestation—NO_2_

Similar findings were reported for exposure to NO_2_ and PTB. A 2011 retrospective cohort study reported a significant positive association per IQR increment of NO_2_ with very PTB in Los Angeles (aOR 1.46; 95% CI: 1.11, 1.92) and Orange County (aOR 1.43; 95% CI: 1.02, 2.01) (Table 5) [29]. A retrospective study in the San Joaquin Valley of California of 252,205 live births by Weber et al. found a slight positive association with NO_2_ exposure during entire pregnancy with PTB at 34–36 weeks among individuals without hypertension (aOR 1.07; 95% CI: 1.04, 1.11 per IQR increase in NO_2_) [35]. Genin et al. reported a positive association of NO_2_ exposure with extreme PTB (RR 1.114; 95% CI: 1.094, 1.135), very PTB (RR 1.046; 95% CI: 1.034, 1.059), and moderate PTB (RR 1.011; 95% CI: 1.006, 1.016) per each unit increase in NO_2_ [30]. In a study investigating PM_2.5_ exposure and proximity to power plants in California, Ha et al. observed higher adjusted odds of PTB (aOR 1.018; 95% CI: 1.013, 1.23) and very PTB (aOR 1.022; 95% CI: 1.010, 1.034) for each 5 km closer residential proximity to a power plant (Table 5) [21].

The same retrospective cohort in Henan Province, China, reported a slight positive association of each standard deviation of total pregnancy NO_2_ exposure with early PTB (aOR 1.682; 95% CI: 1.623, 1.744) and late PTB (aOR 1.593; 95% CI: 1.559, 1.627) (Table 7) [47]. In Tianjin, China, Chen et al. measured associations of exposure to the proportion of days with daily average NO_2_ above the threshold set by the Ambient Air Quality Standards of China (80 μg/m^3^) throughout the entire pregnancy, with PTB length of gestation and reported higher risks of moderate, very, and extreme PTB [45].

### 3.5. Studies Reporting Mixed Findings with Specific PTB Lengths of Gestation—PM_2.5_

A time-stratified case-crossover study of 24,001 singleton live births in Beijing, China measured average exposure during the week before pregnancy and found 5% higher odds of very PTB per 10 µg/m^3^ with no significant associations for moderate PTB (Table 6) [37].

### 3.6. Studies Reporting Mixed Findings with Specific PTB Lengths of Gestation—NO_2_

In a study of 2,928,515 live births in Canada, Stieb et al. reported a positive association of NO_2_ exposure over the entire pregnancy with extreme PTB but null associations with PTB later in gestation (Table 5) [48]. A cohort of 23,086 births in Scotland reported higher risk of very PTB (RR 1.013; 95% CI: 1.00–1.03), but not moderate PTB, per 1 µg/m^3^ of NO_2_ residential exposure [34]. Mendola et al. investigated associations of PM_2.5_ and Nitrogen Oxides (NOx) exposure throughout pregnancy with early PTB < 34 weeks’ gestation and found associations with early PTB among pregnant individuals with asthma (aOR 1.11; 95% CI: 1.01, 1.22) per IQR of PM_2.5_, but a null association among pregnant individuals without asthma. For NOx exposure, the authors reported lower odds of early PTB per IQR of exposure throughout pregnancy (aOR 0.84; 95% CI: 0.79, 0.90) [32].

### 3.7. Studies Reporting Null Findings of PM_2.5_ and NO_2_ with Specific PTB Lengths of Gestation

Several studies reported null findings for the association of PM_2.5_ with PTB length of gestation. Arroyo detected no association of PM_2.5_ or NO_2_ with very PTB or extreme PTB in a study of 298,705 live births in Madrid [27]. In a study of 103,961 singleton births in Florida, Salihu et al. reported a null association of PM_2.5_ exposure with very PTB (Table 4) [24].

A study of 196,780 singleton births in the Huai River Basin of China found no significant associations of PM_2.5_ exposure with moderate PTB or very PTB (Table 6) [38]. Guo et al. reported null associations between preconception PM_2.5_ exposure and extreme PTB, moderate PTB, and very PTB in a birth cohort of 10,916 infants in Tianjin, China [40].

### 3.8. Studies Reporting Both PTB Phenotype and Specific PTB Lengths of Gestation

There were just three studies that analyzed both PTB phenotype and specific lengths of gestation [14,15,16]. A study in New South Wales, Australia of 1,318,570 births found that longer-term exposure to PM_2.5_ was associated with sPTB (HR 1.07; 95% CI: 1.02, 1.12) (Table 1), extreme sPTB (HR 1.34; 95% CI: 1.10, 1.64) and very sPTB (HR 1.20; 95% CI: 1.02, 1.43) per 5 µg/m^3^ of PM_2.5_, while no significant association was detected for moderate to late sPTB (Table 4) [14]. In a study of 285,294 singleton births in New York City, Johnson et al. found no association of first or second trimester PM_2.5_ exposure with sPTB or mPTB and no association of PM_2.5_ with early PTB. Similarly, the authors reported no significant association of first or second trimester NO_2_ exposure with early sPTB or early PTB; however, the authors reported a significant negative association of second trimester NO_2_ exposure with sPTB [15].

In a study of 19,900 singleton births in Shanghai, China, Jiang et al. found no significant association of whole-pregnancy PM_2.5_ with sPTB (Table 3) [16]. However, they observed higher odds of sPTB for third trimester exposures (aOR 1.53; 95% CI: 1.17, 2.01) per IQR of PM_2.5_. The authors also reported a significant association of PM_2.5_ exposure throughout the entire pregnancy with early PTB (aOR 1.80; 95% CI: 1.47, 2.19) and late PTB (aOR 1.35; 95% CI: 1.18, 1.55) per IQR of PM_2.5_, with significant associations also existing for first, second, and third trimester PM_2.5_ exposures with early and late PTB (Table 6).

## 4. Discussion

Among the 134 studies linking PM_2.5_ or NO_2_ to PTB, fewer than one-third addressed either phenotype or specific lengths of gestation. Studies of PTB phenotype are exceedingly rare; just five (3.7%) specify examining either sPTB or mPTB. Findings varied among studies that examined phenotype or specific lengths of gestation; while often positive, some have mixed results and others are null. Results likely vary from differences in exposure timing and level, outcome definition, population, covariates included, and statistical approach. One included study examined twins, a population with inherently higher preterm birth risk; moreover, chorionicity, an important determinant of PTB risk among twins, was not reported, limiting interpretability of these results [12]. Given the extent of heterogeneity across studies in exposure timing, magnitude, and measurement, as well as in outcome definitions and analytic methods, a formal meta-analysis would not be interpretable, as it would synthesize data that are not directly comparable.

### 4.1. Implications of Dearth of Information Regarding PTB Phenotype

While a fair number of studies examine specific lengths of gestation, few examined the reason for PTB. There are likely several reasons for the dearth of data in this area.

One major challenge in investigating air pollution and PTB is the difficulty of phenotyping PTB. PTB is not a singular entity—it arises from diverse and often overlapping pathophysiologic pathways [46,49,50,51,52,53,54]. Broadly, PTB can be categorized as spontaneous, resulting from spontaneous labor or preterm rupture of membranes, or medically indicated, typically due to maternal or fetal conditions such as preeclampsia or fetal growth restriction [53,54,55]. However, in practice, clear-cut differentiation is not always feasible. A pregnancy complicated by fetal growth restriction may lead to mPTB, but that same mother might also be at higher risk for spontaneous labor. Similarly, hypertensive disorders may co-exist with spontaneous membrane rupture, blurring categorical lines.

Studies rarely include standardized or validated algorithms for phenotype assignment and often rely on available administrative codes or chart abstraction with varying degrees of granularity and accuracy [56]. This heterogeneity introduces substantial variability into phenotypic outcome classification [57,58]. By aggregating all PTB cases into a single outcome, most studies risk obscuring important distinctions in biological mechanisms and susceptibility to environmental exposures. If air pollution is associated with specific PTB phenotypes, aggregation could dilute true associations or mask them entirely. Conversely, combining phenotypes with different risk factors and confounders (e.g., socioeconomic status, access to prenatal care, or smoking) may introduce spurious associations, thereby compromising causal inference. Precision in phenotyping is essential to advance mechanistic understanding and to design targeted public health interventions. Without refined outcome definitions, we risk both misclassification and missed opportunities to improve pregnancy outcomes.

A wide range of factors influence the likelihood of sPTB, including genetic predisposition, maternal health conditions, and lifestyle behaviors [55]. Even without fully elucidating the mechanisms of sPTB, identifying associations with air pollution exposure remains critical for informing population-level prevention strategies. In contrast, mPTB is most commonly prompted by conditions such as severe preeclampsia and fetal growth restriction—vascular complications that may themselves be influenced by air pollution. For example, in a study of 34,705 singleton births in Pittsburgh, PA, Lee et al. reported associations between PM_2.5_ exposure and both preeclampsia and gestational hypertension [59]. Urban residence, often linked to higher air pollution levels, along with exposures to other stressors associated with urban life in the U.S., may increase the risk of hypertensive disorders of pregnancy and, in turn, the likelihood of mPTB. Moreover, chronic conditions such as pre-pregnancy hypertension and obesity, which also elevate mPTB risk, may be exacerbated by long-term pollution exposure [60,61].

### 4.2. Reasons for Variation in Findings in Studies That Examine PTB Phenotype or Specific Lengths of Gestation

#### 4.2.1. Duration and Timing of Exposure

Across studies, the timing of air pollution exposure varied considerably, with associations assessed over diverse intervals ranging from short-term (e.g., days prior to delivery), to trimester-specific windows, to entire pregnancy averages. This heterogeneity reflects both differing methodological approaches (e.g., time-series analyses, case–control, ecological regression, logistic regression, Cox regression, Poisson regression, etc.) and underlying hypotheses about when the maternal–fetal dyad may be most vulnerable. While early pregnancy may represent a critical window for placentation and fetal programming, late gestation may be more relevant for triggering labor [62]. Importantly, not all studies align regarding which trimester confers the highest risk, and findings often diverge by pollutant, population, and outcome definition. These inconsistencies highlight the complexity of understanding exposure timing with PTB risk. Moreover, pollution metrics also differ: some report IQR increment, others per 10 µg/m^3^, and some use standardized z-scores. These discrepancies further complicate meta-analytic synthesis and interpretation of effect sizes. This heterogeneity underscores the need for standardized, granular assessments to identify critical periods to clarify potential mechanisms. Future work should also consider how differences in exposure intervals, population demographics, and PTB classification may shape observed associations.

#### 4.2.2. Difference in Air Pollution Exposures and Shape of the Relationship

Ambient PM_2.5_ and NO_2_ exposures vary across the world [63,64]. Huang et al. highlight this, as the annual mean PM_2.5_ was less than 15 µg/m^3^ in the United States in 2015, while it was 58.4 µg/m^3^ in China [65]. This heterogeneity in exposure level may explain variability in results across studies. When interpreting the findings of the included studies, the relationship of PM_2.5_ with PTB is often modeled assuming a linear relationship, but exposure to pollutants at extremely high concentrations likely does not cause the same biological effect as pollutants exposure at low concentrations. In addition, the pollution burden differs substantially by location. Studies conducted in regions with higher concentrations of PM_2.5_ or NO_2_ may observe different dose–response relationships compared to studies in cleaner air environments. Furthermore, PM_2.5_ is highly heterogeneous in terms of pollution source and thus composition and PM_2.5_ species may vary with respect to their biologic impacts. For example, PM_2.5_ composition varies by geographic region in the United States with heavy metals in major urban metropolitan areas from traffic and industrial sources, nitrates and sulfates from coal emissions in Pennsylvania, and organic and elemental carbon from wildfire smoke in the West, while in China PM_2.5_ is sulfate-heavy from coal-based emissions [66,67,68,69,70]. Therefore, results of studies conducted in countries with high pollution burden, such as China, may not be generalizable to the United States. The inclusion of different countries and pollutant exposures complicates the interpretation of exposure–outcome relationships.

#### 4.2.3. Spatial Units

The spatial resolution at which exposure is assigned plays a crucial role in accuracy and inference. Some studies assign exposure based on large administrative regions (e.g., counties, ZIP codes), which may mask significant within-area heterogeneity. Others use fine-scale exposure surfaces (e.g., 1 × 1 km grids or address-level modeling), offering more precise estimation of ambient exposure but often limited to urban or high-resource settings. However, this should be interpreted with nuance as a 1 × 1 km exposure grid has different implications for PM_2.5_ compared to NO_2_ [71,72].

Moreover, exposure is generally assigned based on the maternal residence at delivery, which may not reflect the residential location across all trimesters or account for time spent in other environments (e.g., workplaces, community settings, stores, on vacation). This introduces potential exposure misclassification, especially for time-varying or transient populations. In addition, people often change residences during pregnancy and studies rarely incorporate residential mobility data, despite its relevance for air pollution exposure.

#### 4.2.4. Population and Analytic Approach

Analytical methods used to study air pollution and PTB also vary considerably. Some studies apply individual-level multivariable logistic regression or Cox proportional hazards models, adjusting for sociodemographic and clinical confounders. Others employ spatial ecological models, such as Bayesian Poisson regressions, especially when individual-level data are unavailable. Time-stratified case-crossover designs were used to study short-term effects of air pollution spikes near delivery while limiting confounding [73,74,75].

Outcome definitions also varied across studies. Some studies used a binary definition of PTB (<37 weeks), while others stratified outcomes by gestational age bands (e.g., <28 weeks, 28–32 weeks, 32–36 weeks). Few studies treated gestational age as a continuous variable. These distinctions are important, as risk factors and biological plausibility may differ across the gestational age spectrum [6,76]. Furthermore, few studies examined temporal trends in PTB risk in the context of changing pollution levels or health policies. The use of numerous modeling strategies—coupled with differing definitions of exposure and outcome—complicates efforts to synthesize findings and draw overarching conclusions about the association of PM_2.5_ and NO_2_ exposure with PTB phenotype and length of gestation.

Another source of heterogeneity across studies may stem from live birth bias. Many investigations of air pollution and PTB include only live births, excluding fetal losses or stillbirths that may themselves be related to higher pollution exposure [77]. If exposure increases the risk of pregnancy loss before viability, the remaining live birth cohort may represent a healthier or less susceptible subset, resulting in associations that do not capture all of the risk or accurately quantify effect sizes. This selection bias is especially concerning when exposure timing or intensity affects both early fetal survival and gestational length. Addressing live birth bias through designs that incorporate fetal loss data or sensitivity analyses will improve causal inference in future studies. Specific recommendations to enhance the rigor of studies focused on pollution and PTB are presented in the Supplementary Materials.

### 4.3. Conclusion and Implications for Future Research and Public Health

Although many studies report associations of air pollution with PTB, findings vary because of differences in study design, exposure context, and outcome definition. Few studies of common pollutants such as PM_2.5_ and NO_2_ have examined specific PTB phenotypes (sPTB or mPTB). Lack of outcome specificity limits the understanding of potential mechanisms by which pollution may shorten gestation. Importantly, associations could be missed or diluted. Furthermore, without understanding of pollutants’ associations with distinct phenotypes, targeted exposure mitigation efforts for communities at varying risk for these distinct outcomes is difficult and limits personalized and community-specific public health prevention strategies. Future work to delineate pollution’s contribution to specific PTB phenotypes will enhance efforts to improve birth outcomes around the globe.

## Figures and Tables

**Table 1:** Studies of PM_2.5_ with PTB phenotype as an outcome in North America, Europe, and Australia.

Study	Pollutant	Sample Size (Preterm Cases/Overall *n*)	Setting and Study Type	Exposure Increment	Outcomes: sPTB Estimate (95% CI)	Outcomes: mPTB Estimate (95% CI)
Melody et al., 2020 [9]	PM_2.5_	18,457/285,595	Victoria, Australia 2012–2015, Retrospective Cohort	Per IQR of annual exposure (1.3 µg/m^3^), median 7.1 µg/m^3^	RR 1.04 (1.01, 1.07)	
Gat et al., 2021 [10]	PM_2.5_	5026/63,027	Beersheba Israel 2003–2013, Retrospective Cohort	Per IQR (3.8 µg/m^3^), median 20.3	Jewish population RR 1.014 (0.979, 1.050) Bedouin population RR 1.005 (0.976, 1.035)	
Singh et al., 2023 [14]	PM_2.5_	38,900/1,354,919	New South Wales, Australia 2001–2019, Retrospective Cohort	Per 5 µg/m^3^, median 6.8 µg/m^3^	HRs ranging from 0.86 (0.84, 1.4) to 8.98 (0.97, 1.00) for sPTB overall, extremely sPTB, very sPTB, moderate-to-late sPTB	
Johnson et al., 2016 [15]	PM_2.5_	19,013/258,294	New York City 2008–2010, Retrospective Cohort	Per 10 µg/m^3^, median 11.5 µg/m^3^ for first trimester and 11.4 µg/m^3^ for second trimester	First trimester exposure OR 0.99 (0.90, 1.08), no association with sPTB < 32 weeks’ GA Second trimester exposure OR 0.99 (0.90, 1.09), no association with sPTB < 32 weeks’ gestation	First trimester exposure OR 0.94 (0.84, 1.06) Second trimester exposure OR 0.95 (0.84, 1.08)

Abbreviations: sPTB, spontaneous preterm birth; mPTB, medically indicated preterm birth; IQR, interquartile range; RR, relative risk; HRs, hazard ratios; OR, odds ratio.

**Table 2:** Studies of NO_2_ with PTB phenotype as an outcome in North America, Europe, and Australia.

Study	Pollutant	Sample Size (Preterm Cases/Overall *n*)	Setting and Study Type	Exposure Increment	Outcomes: sPTB Estimate (95% CI)	Outcomes: mPTB Estimate (95% CI)
Melody et al., 2020 [9]	NO_2_	18,457/285,595	Victoria, Australia 2012–2015, Retrospective Cohort	Per IQR of annual exposure (3.9 ppb), median 5.6 ppb	RR 0.93 (0.90, 0.96)	
Escoto et al., 2022 [11]	NO_2_	1708/19,169	Philadelphia, PA 2013–2017, Retrospective Cohort	Per standard deviation (2.3 ppb), mean 17.2 ppb	aOR 0.97 (0.90, 1.05)	aOR 0.97 (0.89, 1.06)
Johnson et al., 2016 [15]	NO_2_	19,013/258,294	New York City 2008–2010, Retrospective Cohort	Per 20 ppb, median 27.1 for first trimester and 26.1 for second trimester	First trimester exposure OR 0.94 (0.87, 1.02), no association with sPTB < 32 weeks’ gestation Second trimester exposure sPTB OR 0.90 (0.83, 0.97), no association with sPTB < 32 weeks’ gestation	First trimester exposure OR 0.90 (0.81, 0.99) Second trimester exposure OR 0.89 (0.80, 0.99)

Abbreviations: sPTB, spontaneous preterm birth; mPTB, medically indicated preterm birth; IQR, interquartile range; RR, relative risk; aOR, adjsuted odds ratio; OR, odds ratio.

**Table 3:** Studies of PM_2.5_ with PTB phenotype as an outcome in China.

Study	Pollutant	Sample Size (Preterm cases/Overall *n*)	Setting and Study Type	Exposure Increment	Outcomes: sPTB Estimate (95% CI)	Outcomes: mPTB Estimate (95% CI)
Qiao et al., 2022 [12]	PM_2.5_	764/ 1515 pairs of twins	Shanghai, China 2013– 2016, Population-based Cohort (retrospective)	Per IQR (4.9 µg/m^3^), median 52.5 µg/m^3^ for entire pregnancy and 52.5 µg/m^3^ for second trimester	Entire pregnancy exposure aOR 0.95 (0.81, 1.11) Second trimester exposure aOR 1.48 (1.06, 2.05)	
Su et al., 2023 [13]	PM_2.5_	9976/ 179,385	Shanghai, China 2014– 2020, Time-series analysis and Retrospective Cohort	Per 10 µg/m^3^, mean 43.2 µg/m^3^	First trimester exposure aOR 0.987 (0.970–1.003) Second trimester exposure aOR 0.993 (0.971–1.016) Third trimester exposure aOR 1.042 (1.018–1.065)	First trimester exposure aOR 0.995 (0.968, 1.023) Second trimester exposure aOR 1.012 (0.981, 1.044) Third trimester exposure aOR 1.022 (0.993, 1.051)
Jiang et al., 2023 [16]	PM_2.5_	1146/ 19,900	Shanghai, China 2015–2017, Retrospective Cohort	Per IQR (9.6 µg/m^3^), median 49.0 µg/m^3^ for entire pregnancy	Entire pregnancy exposure aOR 1.18 (0.98, 1.42) First trimester exposure aOR 1.15 (0.89, 1.48) Second trimester exposure aOR 1.11 (0.97, 1.27) Third trimester exposure aOR 1.53 (1.17, 2.01)	Entire pregnancy exposure aOR 1.37 (1.10, 1.69) First trimester exposure aOR 1.29 (0.95, 1.75) Second trimester exposure aOR 1.21 (1.04, 1.41) Third trimester exposure aOR 1.91 (1.37, 2.69)

Abbreviations: sPTB, spontaneous preterm birth; mPTB, medically indicated preterm birth; IQR, interquartile range; aOR, adjusted odds ratio.

**Table 4:** Studies of PM_2.5_ with PTB specific lengths of gestation in North America, Europe, and Australia.

Study	Pollutant	Sample Size (Preterm cases/Overall *n*)	Setting and Study Type	Exposure Increment	Outcomes: Very or Extreme PTB Estimate (95% CI)	Outcomes: Moderate PTB Estimate (95% CI)	Outcomes: Late PTB Estimate (95% CI)
Ha et al., 2022 [17]	PM_2.5_	44,565/ 196,970	San Joaquin Valley, CA 2007–2015, Time-stratified case-crossover study	Per IQR (15.4–16.1 µg/m^3^), mean 16.8–17.1 µg/m^3^ in week prior to delivery	*Very PTB* Associated with 5–6% increased odds beginning at lag 3: OR 1.06 (1.02, 1.11)		
Mekonnen et al., 2021 [18]	PM_2.5_	87,495/ 953,951	California, USA 2007–2011, Retrospective Cohort	Per 1 µg/m^3^, mean 13.5 µg/m^3^ for entire pregnancy	*Early PTB (<34 weeks)* Entire pregnancy exposure aOR 0.96 (0.92, 1.00) First trimester exposure aOR 0.95 (0.91, 0.99) Second trimester exposure aOR 1.01 (0.97, 1.05) Third trimester exposure aOR 0.91 (0.87, 0.96)		
Sheridan et al., 2019 [19]	PM_2.5_	187,275/ 2,293,218	California, USA 2005–2010, Retrospective Cohort	Per 10 µg/m^3^, mean 13.5 µg/m^3^ for entire pregnancy	*Very PTB* Entire pregnancy exposure aHR 1.19 (1.14, 1.25) First trimester exposure aHR 0.99 (0.96, 1.02) Second trimester exposure aHR 1.08 (1.04, 1.12) Third trimester exposure aHR 1.15 (1.11, 1.19)	Entire pregnancy exposure aHR 1.11 (1.09, 1.14) First trimester exposure aHR 1.00 (0.99, 1.02) Second trimester exposure aHR 1.05 (1.03, 1.06) Third trimester exposure aHR 1.04 (1.03, 1.06)	
Basu et al., 2017 [20]	PM_2.5_	23,265/ 231,637	California, USA 2000–2006, Retrospective Cohort	Per IQR (6.96 µg/m^3^), mean 18.8 µg/m^3^	12–25% increased odds for late, moderate, and very preterm deliveries. No significant association for extremely preterm deliveries.		
Ha et al., 2015 [21]	PM_2.5_	39,082/ 423,719	Florida, USA 2004–2005, Retrospective Cohort	Per 5 km closer residential proximity to any power plant, mean 5.1–17.6 µg/m^3^	*Very PTB* aOR 1.022 (1.010, 1.034)		
Ha et al., 2014 [22]	PM_2.5_	39,082/ 423,719	Florida, USA 2004–2005, Retrospective Cohort	Per IQR (2.6 µg/m^3^), median 9.9 µg/m^3^ for entire pregnancy, 9.6 µg/m^3^ for first trimester, 9.8 µg/m^3^ for second trimester, 10.0 µg/m^3^ for third trimester	*Very PTB* Entire pregnancy exposure aOR 1.082 (1.048, 1.117) First trimester exposure aOR 1.063 (1.028, 1.098) Second trimester exposure aOR 1.215 (1.177, 1.253) Third trimester exposure aOR 1.010 (0.972, 1.049)		
Rappazzo et al., 2014 [23]	PM_2.5_	142,151/ 1,781,527	Pennsylvania, Ohio, and New Jersey 2000–2005, Retrospective Cohort	Per 1 µg/m^3^, mean 14.5 µg/m^3^	*PM_2.5_ exposure during the 4th week of gestation* *Extreme PTB* RD 11.8 (−6, 29.2) *Very PTB* RD 46 (23.2, 68.9)	*PM_2.5_ exposure during the 4th week of gestation* RD 61.1 (22.6, 99.7)	*PM_2.5_ exposure during the 4th week of gestation* RD 28.5 (−39, 95.7)
Salihu et al., 2012 [24]	PM_2.5_	9459/ 103,961	Hillsborough County, Florida, USA 2000–2007, Retrospective Cohort	Exposure above median (>11.3 µg/m^3^)	*Very PTB* aOR 1.05 (0.93–1.18)		
Cassidy-Bushrow et al., 2020 [25]	PM_2.5_	891/ 7961	Detroit, MI 2008–2010, Retrospective Cohort	Per 5 µg/m^3^, mean 10.7 µg/m^3^	In the fully adjusted model, PM_2.5_ (mean change in gestational age [weeks]. 0.14 ± 0.12; *p* = 0.255) was not associated with gestational age at delivery.		
Laurent et al., 2016 [26]	PM_2.5_	442,314/ 3,870,696	California, USA 2001–2008, Nested case–control	Per IQR increase (6.5 µg/m^3^), median exposure not reported	*Very PTB* aOR 1.102 (1.071, 1.134)	aOR 1.138 (1.119, 1.156)	
Arroyo et al., 2016 [27]	PM_2.5_	24,620/ 298,705	Madrid, Spain 2001–1009, Time series analysis	Per IQR increase (IQR not reported, mean (SD) 17.1 (7.8) µg/m^3^)	No association of PM_2.5_ with *very preterm birth or extremely preterm birth* from 0 to 7 days preceding birth reported		
Padula et al., 2014 [28]	PM_2.5_	30,963/ 263,204	San Joaquin Valley, CA 2000–2006, Retrospective Cohort	Exposure in the highest quartile (20.8 µg/m^3^) combined to the lowest three quartiles combined, mean 18.0–18.6 µg/m^3^	*Very PTB* aOR 1.62 (1.45–1.81) *Extreme PTB (24–27 weeks)* aOR 1.81 (1.50–2.18) *Extreme PTB (20–23 weeks)* *aOR 1.25 (0.95, 1.65)*	aOR 1.54 (1.41, 1.68)	aOR 1.27 (1.22, 1.32)
Wu et al., 2011 [29]	PM_2.5_	6712/ 81,186	Los Angeles and Orange Counties, California 1997–2006, Retrospective Cohort	Per 1 µg/m^3^, mean 17.3 µg/m^3^	*Very PTB* Los Angeles County aOR 1.03 (0.81–1.30) Orange County aOR 1.33 (0.99–1.77)		
Genin et al., 2022 [30]	PM_2.5_	278,817/ 5,070,262	France 2012–2018, Cross-sectional Study	Ecological regression, relative risk of PTB and 95% credibility interval, median 9.5 µg/m^3^	*Very PTB* RR 1.072 (1.051, 1.094) *Extreme PTB* RR 1.191 (1.153, 1.230)	RR 1.020 (1.012,1.028)	
Singh et al., 2023 [14]	PM_2.5_	38,900/ 1,354,919	New South Wales, Austria 2001–2019, Retrospective Cohort	Per 5 µg/m^3^, median 6.8 µg/m^3^	HRs ranging from 0.86 (0.84, 1.4) to 8.98 (0.97, 1.00) for extremely sPTB, very sPTB, moderate-to-late sPTB		
Johnson et al., 2016 [15]	PM_2.5_	19,013/ 258,294	New York City 2008–2010, Retrospective Cohort	Per 10 µg/m^3^, median 27.1 for first trimester and 26.1 for second trimester	No evidence of association of first or trimester exposure with early preterm birth.		
Studies investigating effect modification by disease process or birthing parent comorbidity on relationship of PM_2.5_ with PTB length of gestation							
Padula et al., 2019 [31]	PM_2.5_	28,788/ 252,205	San Joaquin Valley, CA 2000–2006, Retrospective Cohort	Exposure in the highest quartile (17.1 µg/m^3^) combined to the lowest three quartiles combined, median 17.1 µg/m^3^	*Very PTB* Without diabetes aOR 1.37 (1.25, 1.50) With diabetes aOR 1.27 (0.89, 1.81) *Extreme PTB* Without diabetes aOR 1.58 (1.40, 1.78) With diabetes aOR 2.44 (1.39, 4.29)	Without diabetes aOR 1.46 (1.36, 1.58) With diabetes aOR 1.35 (1.03, 1.76)	Without diabetes aOR 1.23 (1.19, 1.27) With diabetes aOR 1.19 (1.05, 1.34)
Mendola et al., 2016 [32]	PM_2.5_	26,144/ 223,502	US 2002–2008 Retrospective Cohort	Per IQR (4.7 µg/m^3^), median 11.9 µg/m^3^	*Preterm birth < 34 weeks’ gestation among birthing parents with asthma* aOR 1.11 (1.01, 1.22)		

Abbreviations: Extreme PTB, <28 weeks; Very PTB, 28–31 weeks; Moderate PTB, 32–33 weeks; Late PTB, 34–36 weeks; aOR, adjusted odds ratio; aHR, adjusted hazard ratio; HR, hazard ratio; RD, risk difference; IQR interquartile range; SD, standard deviation.

**Table 5:** Studies of NO_2_ or NO_x_ with PTB specific lengths of gestation in North America and Europe.

Study	Pollutant	Sample Size (Preterm cases/Overall *n*)	Setting and Study Type	Exposure Increment	Outcomes: Very or Extreme PTB Estimate (95% CI)	Outcomes: Moderate PTB Estimate (95% CI)	Outcomes: Late PTB Estimate (95% CI)
Stieb et al., 2016 [33]	NO_2_	182,475/ 2,928,515	Canada 1999–2008, Retrospective Cohort	Per IQR (11.5 ppb), median 11.9 ppb	*Extreme PTB* OR 1.10 (1.05, 1.15) *Very PTB* OR 1.00 (0.96, 1.04)	OR 1.00 (0.97, 1.03)	OR 0.97 (0.96, 0.98)
Cassidy-Bushrow et al., 2020 [25]	NO_2_	891/ 7961	Detroit, MI 2008–2010, Retrospective Cohort	Per 5 ppb, median 18.5 ppb	In the fully adjusted model, NO_2_ (mean change in gestational age [weeks] 0.11 ± 0.09; *p* = 0.245) was not associated with gestational age at delivery		
Laurent et al., 2016 [26]	NO_2_	442,314/ 3,870,696	California, USA 2001–2008, Nested case–control	Per IQR increase (10.0 ppb), median exposure not reported	*Very PTB* aOR 1.048 (1.018, 1.080)	aOR 1.077 (1.061, 1.094)	
Arroyo et al., 2016 [27]	NO_2_	24,620/ 298,705	Madrid, Spain 2001–2009, Time series analysis	Per IQR increase (IQR not reported, mean (SD) 59.4 (17.9) µg/m^3^)	No association of NO_2_ with *very PTB* or *extreme PTB* from 0 to 7 days preceding birth reported		
Padula et al., 2014 [28]	NO_2_	30,963/ 263,204	San Joaquin Valley, CA 2000–2006, Retrospective Cohort	Exposure in the highest quartile (19.5 ppb) combined to the lowest three quartiles combined, mean exposure 16.8–17.7 ppb	*Very PTB* aOR 1.13 (1.00, 1.27) *Extreme PTB (24–27 weeks)* aOR 1.08 (0.88, 1.33) *Extreme PTB (20–23 weeks)* aOR 0.88 (0.51, 1.17)	aOR 1.13 (1.03, 1.25)	aOR 1.11 (1.06, 1.15)
Wu et al., 2011 [29]	NO_2_	6712/81,186	Los Angeles and Orange Counties, California 1997–2006, Retrospective Cohort	Per 5 ppb increase, mean 24.9 ppb	*Very PTB* Los Angeles County aOR 1.46 (1.11, 1.92) Orange County aOR 1.43 (1.02, 2.01)		
Genin et al., 2022 [30]	NO_2_	278,817/5,070,262	France 2012–2018, Cross-sectional study	Ecological regression, relative risk of PTB and 95% credibility interval, median 8.1 µg/m^3^	*Very PTB* RR 1.046 (1.035, 1.059) *Extreme PTB* RR 1.114 (1.094, 1.135)	RR 1.011 (1.006, 1.016)	
Dibben et al., 2015 [34]	NO_2_	1242/ 23,086	Scotland 1994–2018, Retrospective Cohort	Per 1 µg/m^3^, mean 17.5 µg/m^3^	*Very PTB* aOR 1.013 (1.00, 1.03)	aOR 1.00 (0.99, 1.00)	
Johnson et al., 2016 [15]	NO_2_	19,013/ 258,294	New York City 2008–2010, Retrospective Cohort	Per 20 ppb, median 27.1 for first trimester and 26.1 for second trimester	No evidence of association of first or trimester exposure with early preterm birth.		
Studies investigating effect modification of disease process or birthing parent comorbidity on relationship of PM_2.5_ with PTB lengths of gestation.							
Mendola et al., 2016 [32]	NO_x_	26,144/223,502	US 2002–2008 Retrospective Cohort	Per IQR (24.2 ppb), median 30.9 ppb	No significant association of preterm birth < 34 weeks’ gestation among birthing parents with or without asthma		
Weber et al., 2019 [35]	NO_2_	28,788/252,205	San Joaquin Valley, CA 2000–2006, Retrospective Cohort	Exposure in the highest quartile (19.5 ppb) combined to the lowest 3 quartiles combined, median 17.3	*Very PTB* Without hypertension aOR 1.13 (1.03, 1.24) With hypertension aOR 0.99 (0.78, 1.26) *Extreme PTB* Without hypertension aOR 1.21 (1.06, 1.37) With hypertension aOR 1.49 (1.00, 2.21)	Without hypertension aOR 1.11 (1.02, 1.20) With hypertension aOR 1.16 (0.96, 1.41)	Without hypertension aOR 1.07 (1.04, 1.11) With hypertension aOR 1.04 (0.94, 1.16)
Padula et al., 2019 [31]	NO_2_	28,788/252,205	San Joaquin Valley, CA 2000–2006, Retrospective Cohort	Exposure in the highest quartile (17.3 ppb) combined to the lowest three quartiles combined, median 17.3 ppb	*Very PTB* Without diabetes aOR 1.12 (1.02, 1.23) With diabetes aOR 0.92 (0.64, 1.33) *Extreme PTB* Without diabetes aOR 1.21 (1.07, 1.37) With diabetes aOR 1.56 (0.87, 2.80)	Without diabetes aOR 1.12 (1.03, 1.21) With diabetes aOR 1.04 (0.79, 1.37)	Without diabetes aOR 1.07 (1.04, 1.11) With diabetes aOR 0.99 (0.87, 1.12)

Abbreviations: Extreme PTB, <28 weeks; Very PTB, 28–31 weeks; Moderate PTB, 32–33 weeks; Late PTB, 34–36 weeks; aOR, adjusted odds ratio; RR, relative risk; IQR interquartile range; SD, standard deviation.

**Table 6:** Studies of PM_2.5_ with PTB specific lengths of gestation as an outcome in China.

Study	Pollutant	Sample Size (Preterm cases/Overall *n*)	Setting and Study Type	Exposure Increment	Outcomes: Very or Extreme PTB Estimate (95% CI)	Outcomes: Moderate PTB Estimate (95% CI)	Outcomes: Late PTB Estimate (95% CI)
He et al., 2022 [36]	PM_2.5_	287,433/ 3,723,169	China 2010–2015, Retrospective Cohort	Per IQR (29 µg/m^3^), median 54 µg/m^3^	*Very PTB* HR 1.02 (1.00, 1.04)	HR 1.06 (1.04, 1.08)	HR 1.10 (1.08, 1.11)
Fang et al., 2022 [37]	PM_2.5_	1062/ 24,001	Beijing, China 2014–2017, Retrospective Cohort	Per 10 µg/m^3^, mean 85.9 µg/m^3^ for first trimester, 84.9 µg/m^3^ for second trimester, and 82.7 µg/m^3^ for third trimester	*Very PTB* First trimester exposure aOR 0.90 (0.72, 1.13) Second trimester exposure aOR 0.98 (0.76, 1.26) Third trimester exposure aOR 3.31 (2.46, 4.46)	First trimester exposure aOR 0.97 (0.91, 1.03) Second trimester exposure aOR 1.00 (0.92, 1.09) Third trimester exposure aOR 1.90 (1.74, 2.08)	
Zhang et al. 2020 [44]	PM_2.5_	273/ 2101	Wuhan City, China 2013–2015, Retrospective Cohort	Per 10 µg/m^3^, mean 84.5 µg/m^3^	*Very PTB* Entire pregnancy exposure aOR 1.496 (1.222, 1.778) First trimester aOR 1.265 (1.116, 1.417) Second trimester aOR 1.111 (1.005, 1.218) Third trimester aOR 1.054 (0.925, 1.186)	Entire pregnancy exposure aOR 1.230 (1.18, 1.344) First trimester aOR 1.170 (1.071, 1.269) Second trimester aOR 1.051 (1.008, 1.094) Third trimester aOR 1.053 (1.000, 1.106)	
Guo et al. 2018 [39]	PM_2.5_	35,261/ 426,246	China 2014, Retrospective Cohort	Per 10 µg/m^3^, median 57.9 µg/m^3^ for entire pregnancy, 57.9 µg/m^3^ for first trimester, 60.3 µg/m^3^ for second trimester, 52.3 µg/m^3^ for third trimester	*Very PTB* Entire pregnancy exposure aHR 1.12 (1.11, 1.14) First trimester exposure aHR 1.07 (1.06, 1.08) Second trimester exposure aHR 1.05 (1.04, 1.06) Third trimester exposure aHR 1.11 (1.10, 1.12)		
Guo et al. 2023 [40]	PM_2.5_	237/ 10,916	Tianjin, China, Retrospective Cohort	By exposure higher than median (75 µg/m^3^) during oogensis and spermatogenesis	No statistically significant association between PM_2.5_ exposure during both oogenesis and spermatogenesis and any preterm birth, moderate PTB, very PTB, or extreme PTB.		
Qiu et al. 2023 [41]	PM_2.5_	82,820/ 2,294,188	China 2013–2019, Retrospective Cohort	Per 10 µg/m^3^, mean 52.5 µg/m^3^	*Very PTB* aOR 2.06 (1.95, 2.17)	aOR 1.28 (1.26, 1.30)	
Zhou, 2022 [42]	PM_2.5_	15,224/ 697,316	Henan Province, China 2014–2016, Retrospective Cohort	Per SD (11.7–27.6 µg/m^3^), mean 72.3 µg/m^3^ for entire pregnancy, 71.1 µg/m^3^ for first trimester, 72.3 µg/m^3^ for second trimester, and 73.5 µg/m^3^ for third trimester	*Early PTB (<34 weeks)* Entire pregnancy exposure OR 1.682 (1.623, 1.744) First trimester exposure OR 0.898 (0.840, 0.961) Second trimester exposure OR 1.061 (0.988, 1.139) Third trimester exposure OR 1.353 (1.261, 1.452)		*Late PTB (34–36 weeks)* Entire pregnancy exposure OR 1.593 (1.559, 1.627) First trimester exposure OR 1.102 (1.060, 1.146) Second trimester exposure OR 1.217 (1.166, 1.270) Third trimester exposure OR 1.228 (1.180, 1.279)
Chen, 2021 [45]	PM_2.5_	241/ 10,960	Tianjin, China, 2014–2016, Retrospective Cohort	Per 5 µg/m^3^, mean 55 µg/m^3^	*Very PTB* HR 3.52 (2.42, 5.13) *Extreme PTB* HR 2.11 (1.83, 2.44)	HR 2.06 (1.68, 2.52)	
Wang, 2018 [46]	PM_2.5_	25,879/ 469,975	Guangzhou, China 2015–2017, Retrospective Cohort	Per IQR increase (27 µg/m^3^), median 34 µg/m^3^	*Very PTB* HR 1.671 (0.423, 6.596)	HR 0.982 (0.580, 1.663)	
Jiang et al. 2023 [16]	PM_2.5_	1146/ 19,900	Shanghai, China 2015–2017, Retrospective Cohort	Per IQR (9.6 µg/m^3^), median 49.0 µg/m^3^ for entire pregnancy	*Early PTB (<34 weeks’ gestation* Entire pregnancy exposure aOR 1.80 (1.47, 2.19) First trimester exposure aOR 1.58 (1.20, 2.08) Second trimester exposure aOR 1.64 (1.42, 1.89) Third trimester exposure aOR 3.36 (2.45, 4.64)		
Studies investigating effect modification by temperature							
Zhang et al. 2023 [38]	PM_2.5_	4257/ 196,780	Huai River Basin, China 2013–2018, Retrospective Cohort	Exposure above Median (>77.5–95.1 µg/m^3^)	*In extreme heat, very PTB:* Second trimester OR 2.841 (1.910, 4.224) Third trimester OR 2.117 (1.367, 3.278)	*In extreme heat, very PTB:* First trimester OR 1.290 (1.048, 1.589) Third trimester OR 1.971 (1.631, 2.381)	

Abbreviations: Extreme PTB, <28 weeks; Very PTB, 28–31 weeks; Moderate PTB, 32–33 weeks; Late PTB, 34–36 weeks; aOR, adjusted odds ratio; HR hazard ratio; IQR interquartile range; SD, standard deviation.

**Table 7:** Studies of NO_2_ or NO_x_ with PTB specific lengths of gestation in China.

Study	Pollutant	Sample Size (Preterm cases/Overall *n*)	Setting and Study Type	Exposure Increment	Outcomes: Very or Extreme PTB Estimate (95% CI)	Outcomes: Moderate PTB Estimate (95% CI)	Outcomes: Late PTB Estimate (95% CI)
Zhou et al., 2022 [42]	NO_2_	15,224/697,317	Henan Province, China 2014–2016, Retrospective Cohort	Per SD (8.0–12.0 µg/m^3^), mean 37.5 µg/m^3^ for entire pregnancy, 36.6 µg/m^3^ for first trimester, 37.6 µg/m^3^ for second trimester, 38.1 µg/m^3^ for third trimester	*Early PTB (<34 weeks)* Entire pregnancy exposure OR 1.682 (1.623, 1.744) First trimester exposure OR 1.754 (1.672, 1.840) Second trimester exposure OR 1.917 (1.823, 2.016) Third trimester exposure OR 1.887 (1.797, 1.982)		Entire pregnancy exposure OR 1.593 (1.559, 1.627) First trimester exposure OR 1.713 (1.665, 1.762) Second trimester exposure OR 1.784 (1.730, 1.839) Third trimester exposure OR 1.850 (1.800, 1.901)
Chen et al., 2021 [45]	NO_2_	241/10,960	Tianjin, China 2014–2016, Retrospective Cohort	Per 3 µg/m^3^, mean 41.6 µg/m^3^	*Very PTB* HR 3.26 (2.06,5.14) *Extreme PTB* HR 1.21 (1.06,1.38)	HR 1.56 (1.25,1.94)	
Wang et al., 2018 [46]	NO_2_	25,879/469,975	Guangzhou, China 2015–2017, Retrospective Cohort	Per IQR increase (29 ppb), median 42 ppb	*Very PTB* HR 2.805 (2.037, 3.861)	*Moderate PTB* HR 1.133 (0.969, 1.326)	

Abbreviations: Extreme PTB, <28 weeks; Very PTB, 28–31 weeks; Moderate PTB, 32–33 weeks; Late PTB, 34–36 weeks; OR, odds ratio; HR hazard ratio; IQR interquartile range; SD, standard deviation.

## Data Availability

No new data were created or analyzed in this study. Data sharing is not applicable to this article.

## Supplementary Materials

The following supporting information can be downloaded at: https://www.mdpi.com/article/10.3390/children13010002/s1, Supplemental Table S1. Recommended Reporting Elements for Studies of Air Pollution and Preterm Birth (PTB).

## Abbreviations

The following abbreviations are used in this manuscript:

aHR	adjusted hazard ratio
PTB	preterm birth
sPTB	spontaneous preterm birth
mPTB	medical preterm birth
PM_2.5_	particulate matter
NO_2_	nitrogen dioxide
PRISMA	Preferred Reporting Items for Systematic Reviews and Meta-Analyses
MeSH	Medical Subject Headings
IQR	interquartile range
OR	odds ratio
CI	confidence interval
aOR	association odds ratio
HR	hazard ratio
RR RD	relative risk risk difference
