# Supplementary material for: Air Pollution and Preterm Birth: A Scoping Review Focused on Preterm Birth Phenotype and Specific Lengths of Gestation
Source: Children (Basel). 2025 Dec 19;13(1):2. doi: 10.3390/children13010002 (PMC12840119; doi:10.3390/children13010002)
Supplement: Supplementary file 1 [file children-13-00002-s001.zip › children-4008725-supplementary.pdf]

Supplemental Table S1. Recommended Reporting Elements for Studies of Air Pollution and Preterm Birth (PTB)

Domain	Recommended Minimum Reporting Elements
**PTB Phenotype Definition**	Clear definitions of spontaneous and medically indicated preterm birth; method of phenotype assignment (administrative codes, chart review, validated algorithm); handling of ambiguous or overlapping cases.
**Gestational Age Classification**	Explicit gestational age (GA) assignment (e.g., last menstrual period, ultrasound, best obstetric estimate) and GA cutoffs for extremely preterm, very preterm, moderate preterm, and late preterm; specify whether GA was modeled continuously or categorically.
**Exposure Assessment**	Pollutant type; exposure increments used (e.g., per IQR, per 10 μg/m^3); spatial resolution of exposure surface; temporal window (trimester-specific, weekly, whole-pregnancy).
**Context of Ambient Exposure**	Mean, median, and IQR pollutant concentrations for the study region; comparison to regional or World Health Organization air quality guidelines.
**Covariates**	List of included covariates with justification; description of potential residual confounding; treatment of socioeconomic variables and smoking.
**Residential Mobility**	Whether changes in maternal residence during pregnancy were ascertained or if residential address was assumed static.
**Model Specification**	Statistical model used; assessment of nonlinearity; use of sensitivity analyses (e.g., alternative exposure windows, multi-pollutant models).
**Live Birth Bias / Selection Factors**	Whether fetal losses or stillbirths were considered; discussion of implications for causal inference.
**Effect Modification**	Assessment of effect modification by comorbidities, parity, temperature, or other relevant factors.
